# The Effect of Vibratory Grinding Time on Moisture Sorption, Particle Size Distribution, and Phenolic Bioaccessibility of Carob Powder

**DOI:** 10.3390/molecules27227689

**Published:** 2022-11-09

**Authors:** Libor Červenka, Michaela Frühbauerová, Jiří Palarčík, Sali Muriqi, Helena Velichová

**Affiliations:** 1 Department of Analytical Chemistry, Faculty of Chemical Technology, University of Pardubice, Studentská 573, 532 10 Pardubice, Czech Republic; 2 Institute of Environmental and Chemical Engineering, Faculty of Chemical Technology, University of Pardubice, Studentská 573, 532 10 Pardubice, Czech Republic; 3 Department of Food Analysis and Chemistry, Faculty of Technology, Tomáš Baťa University in Zlín, Nám. T. G. Masaryka 5555, 460 01 Zlín, Czech Republic

**Keywords:** phenolic, flavonoid, HPLC analysis, in vitro digestion, correlation

## Abstract

Carob pod powder, an excellent source of health-promoting substances, has found its use in a wide range of food products. Grinding conditions affect the physical and chemical properties of the powder, but their influence on the bioaccessibility of phenolic compounds in carob pod powder has not yet been determined. The carob pods were ground for 30–180 s in a vibratory grinder. The median values (D_50_) of particle size decreased after 60 s of grinding (87.9 μm), then increased to 135.1 μm. Lightness showed a negative correlation with D_50_ and a_w_, while the values of redness and yellowness decreased with the reduction in particle size and water activity. The smaller the value of D_50_, the higher the equilibrium moisture content of carob powder. Phenolic acids (vanillic, ferulic, cinnamic) and flavonoids (luteolin, naringenin, apigenin) were found in all samples of carob powder. The grinding time influenced their content in carob powder, with maximum values at 180 s. Similar observations were made when assessing antioxidant capacity. The in vitro digestion process only improved the bioaccessibility of catechin content in all samples. However, the bioaccessibility of the phenolic compounds and the total phenolic and flavonoid contents decreased with the increase in grinding time. Our findings revealed that the grinding of carob pods for 180 s improved the extractability of phenolics; however, their bioaccessibility was reduced. It is sufficient to ground the carob pod for 30 s, ensuring good availability of nutraceuticals and lower energy cost for grinding.

## 1. Introduction

The carob pod is the fruit of *Ceratonia siliqua* L., a tree of the family Fabaceae. Carob pods serve as a valuable source of essential nutrients, including protein, essential fatty acids, and calcium [1]. It has a promising composition in relation to human health, containing various types of polyphenols (tannins, flavonoids, phenolic acids, etc.) and fibres [2,3]. In addition to the beneficial effect on metabolic health, carob cultivation in the Mediterranean area has a low carbon footprint, which is useful for achieving sustainable development goals [4]. Plant tissues are usually processed by drying to preserve the content of biologically active substances. Subsequently, the dried material is milled to a powder that can be used in various food formulations. As mentioned in a study by Issaoui et al. [5], the grinding of dried carob is easier compared to the grinding of wheat, resulting in low energy consumption. Their claim was supported by the development of a formula for manufacturing bread in which carob pod powder was used as a partial replacement for wheat flour. Since carob pod is naturally gluten-free, other products have been developed to reduce gluten content, such as muffins [6,7], pasta [8], and cookies [9]. In addition to that, carob powder was added to chocolate [10] or Halva [11] to replace cocoa powder and obtain nutritionally more valuable confectionary products. In terms of the production of value-added food products, processes have been proposed to manufacture alcoholic beverages and carob-based drink powder [12,13]. Regarding the literature published in recent years and consumer demand for healthier food products, carob pod powder is at the forefront of the food industry.

Both drying and grinding techniques have an effect on particle size distribution, powder fluidity, colour, and hydration [14]. The influence of the particle size on the physicochemical properties of plant-based powders has been well documented [15,16,17,18]. However, in these studies, powder fractions with different particle size distributions were obtained by sieving. Several studies were conducted to elucidate the effect of grinding time on the functional properties of powder samples using a planetary ball mill [19,20,21,22,23]. Although particle size decreased with the increase in grinding time in all studies cited, the researchers concluded that some chemical properties of the powders were not proportional to the grinding time. For instance, onion peel powder had a maximum value of total phenolic content (TPC) after 18 h of milling, while a significant decrease was observed after 24 h [20]. Norhidayah et al. determined higher TPC values for the ground ginger rhizome after 2 and 4 h than those obtained after 6 or 8 h of grinding [21]. These findings indicate that a longer grinding time does not necessarily guarantee better powder properties. Therefore, it is advisable to find specific milling conditions for each particular material.

It is known that the decrease in particle size leads to an increase in the surface area of the particles, resulting in better extraction efficiency for various bioactive compounds [24,25,26]. However, the concept of chemical extraction alone is no longer sufficient, and the bioaccessibility of nutrients in relation to the structure of the food product must be examined [27]. A study of the bioaccessibility of phenolics from carob products has been published in only a few articles. Chait et al. [28] found that in vitro digestion affected the amount of free, bound, and conjugated phenolic compounds in carob powder. The three-step digestion process (oral, gastric, and intestinal) strongly reduced the phenolic content and antioxidant capacity of carob pulp [29]. In our recent work, the effect of cryogenic and vibratory grinding on the content of phenolic acids was studied [30]. It is unknown whether different grinding times affect the bioaccessibility of phenolic content and antioxidant potential using a vibratory grinder. We assume that the increase in grinding time positively affects carob powder’s particle size distribution and moisture adsorption properties. Thus, a higher bioaccessibility of phenolics, flavonoids, and catechins is expected.

## 2. Results and Discussion

### 2.1. The Effect of Grinding Time on Particle Size, Colour, and Moisture Adsorption Properties of Carob Powder

Carob powder (CP) was prepared by vibratory grinding at various processing times (30, 60, 90, 120, and 180 s). In the text, these samples are referred to as CP30, CP60, CP90, CP120, and CP180, respectively. The physical parameters are shown in Table 1. The grinding time had a significant effect on the D_50_ values, decreasing significantly from 141.3 ± 2.5 to 87.9 ± 1.7 μm (*p* < 0.001) when ground for 30 and 60 s, respectively. A further increase in the grinding time resulted in a gradual increase in D_50_ values up to 135.1 ± 1.8 μm for CP180. A similar pattern was observed for the D_10_ values. However, the particle size in the upper percentile (D_90_) showed a different behaviour: high D_90_ values of 527.7 ± 8.1 and 810.6 ± 21.3 μm were observed for CP60 and CP120, respectively. These findings also corresponded to high span values showing the lack of distribution uniformity (span 6.3 and 6.1, respectively). It was previously published that grinding time significantly influenced the particle size distribution of various food powders.

Reduction in particle size was observed with the increase of ball milling time for onion peel powder [20], ginger rhizome powder [21], horseradish powder [22], or soybean protein isolate powder [23]. Longer grinding time resulted in an increase in the D_90_ value caused by the stickiness of the particles and the formation of agglomerates for horseradish powder [22]. The agglomeration process occurred during the entire grinding of dried mushrooms and was effectively disrupted by ultrasound treatment [19].

The grinding time of the carob pods significantly influenced all the values that describe the colour of the carob powder (*p* < 0.01). Lightness increased after 60 s of grinding (L* = 51.1 ± 0.4), then gradually decreased with the increase in processing time (Table 1). Carob powder ground for 120 s and 180 s had higher values of redness (a* = 6.8 ± 0.4 and 6.9 ± 0.2, respectively) and yellowness (b* = 20.1 ± 0.4 and 19.8 ± 0.3, respectively).

Different colours can be visually observed only for CP60 in our study. Correlation analysis revealed a strong association between the median particle size (D_50_) and the colour in terms of L* (*r* = −0.961, *p* < 0.001), a* (*r* = 0.634, *p* < 0.01), and b* (*r* = 0.764, *p* < 0.001). Interestingly, all colour values exhibited an association with the water activity of the carob powder. Whereas the lightness showed a negative association with a_w_ (*r* = −0.548, *p* < 0.05), water activity positively correlated to a* (*r* = 0.781, *p* < 0.01) and b* values (*r* = 0.795, *p* < 0.001). The water activity of the carob powder was similar for CP30 and CP60, followed by its increase while increasing grinding time. It might seem that the smaller the particles, the lower the water activity, as can be seen in Table 1; however, the correlation coefficient indicates a weak and negligible association (*r* = 0.464, *p* > 0.05).

Colour changes have been observed during the grinding of flour [6], rose-myrtle powder [17], or black kidney bean powder [18]. Drakos et al. [16] found that the colour changes during grinding were product-specific, and different changes of a* and b* values were determined for rye flour and barley flour prepared by jet-milling. Various fractions of particle size of kidney bean powder exhibited a weak association with colour stimuli; for example, the values of b* decreased with the increase of particle size from 125 to 250 μm but increased significantly with a further reduction in particle diameter [18]. It should be noted that colour changes can also be affected by increasing temperature during milling. The colour of the carob powder was influenced by temperature treatment, including roasting [31] or spray-drying [32]. In this study, the temperature of the metal equipment after 30 and 180 s of grinding was 22–24 °C and 37–39 °C, respectively. According to the IUPAC classification, the shape of the moisture adsorption isotherms of all carob powder samples is type III [33], with an increase of the equilibrium moisture content (EMC) to ~25 mg/g in the range of 0–40% relative humidity (RH) followed by a steep increase to ~259–268 mg g^−1^ in the range of 40 to 80% RH. This is an obvious behaviour of high-sugar food products [34]. The differences in the adsorption isotherm plots for the carob powder samples are barely visible; therefore, we compared the EMC for two levels of RH.

**Figure 1:**
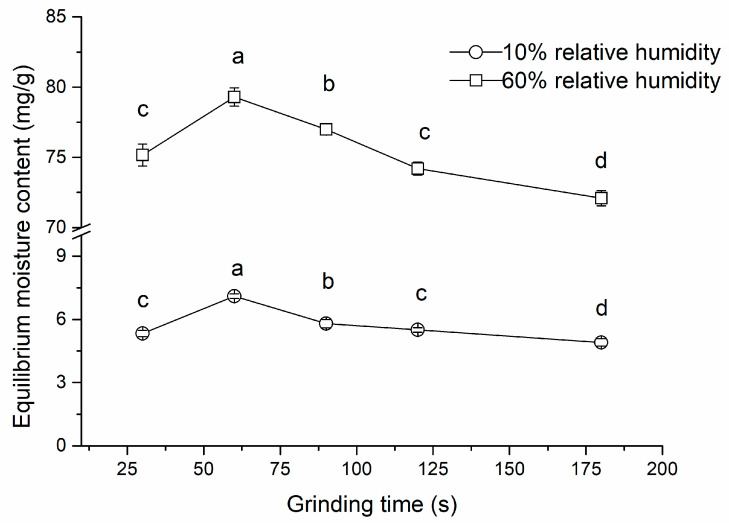
The effect of grinding time on the equilibrium moisture content of carob powder at two relative humidity (RH) levels. Mean ± standard deviation (N = 2). Different small letters indicate significant differences (*p* < 0.05) within the RH level.

As can be seen in Figure 1, the EMC was significantly higher (*p* < 0.05) for the CP60 sample at 10% (7.1 mg g^−1^) and 60% (79.3 mg g^−1^) of RH compared to other grinding times. The EMC decreased with an increase in the grinding time from 60 to 180 s. These findings corresponded to the particle size distribution, where a strong association was found between D_50_ and EMC at 10% (*r* = −0.883, *p* < 0.05) and 60% (*r* = −0.884, *p* < 0.05) of RH. It was well documented that reducing the particle size distribution results in improved water adsorption measured by water holding capacity, i.e., the smaller the particles, the higher the surface area available for moisture uptake [15,24].

### 2.2. Grinding Time as Affected Antioxidant Properties and Phenolic Content in Carob Powder

Twelve phenolic standards were involved in the HPLC analysis. Only 3 phenolic acids (vanillic, ferulic, and cinnamic), two flavons (luteolin and apigenin), and naringenin (flavanone) were identified in carob powder extract (Figure S1). The effect of grinding time on the phenolic content and antioxidant properties is presented in Table 2. ANOVA revealed that the grinding time had a significant effect on all variables (*p* < 0.001), excluding vanillic acid. Cinnamic acid was the most abundant phenolic constituent, with a maximum value of 54.28 ± 1.42 μg g^−1^ in the CP180 sample.

The carob powder ground for 180 s had a significantly higher content of all phenolics determined in this study compared to the CP120 sample. Total phenolic content (TPC) ranged from 4.73 ± 0.21 to 6.07 ± 0.15 GAE/(mg g^−1^), with a maximum value for CP180. Total flavonoid content (TFC) was significantly higher for CP60 (0.33 ± 0.13 QUE/(mg g^−1^)) compared to CP30 (*p* < 0.05). The increase in grinding time from 90 to 120 s resulted in the same TFC values (*p* > 0.05). Although the catechin content (CC) appeared to increase with the increase in grinding time, the differences were not statistically significant (*p* > 0.05). DPPH values increased after 60 s of grinding to 11.91 ± 0.51 TEAC/(mg g^−1^) (*p* < 0.05), then decreased significantly for CP90 (*p* < 0.05) followed by a further increase reaching its maximum value of 11.93 ± 0.74 TEAC/(mg g^−1^) for CP180. The FRAP values followed the same pattern with the highest antioxidant capacity for the CP120 sample (16.95 ± 1.08 TEAC/(mg g^−1^)). As expected, positive associations were found between antioxidant properties in terms of the DPPH and FRAP assays and TPC, TFC, and catechin content (*r* = 0.656–0.836, *p* < 0.05). The particle size of chia seeds was positively associated with the release of phenolic compounds during the extraction procedure [25]. On the contrary, some experiments did not confirm the correlation between particle size and phenolic content [26] or antioxidant capacity [16], probably due to different materials, grinding techniques, or particle size ranges. In our study, a weak negative association (*p* > 0.05) was observed between D_50_ and TPC (*r* = −0.421), ferulic acid (*r* = −0.439), and naringenin (*r* = −0.460) contents. The higher ability to eliminate the DPPH radical was significantly associated with lower D_50_ values (*r* = −0.606, *p* < 0.05). It suggests that small-sized carob powder can release more phenolics during extraction.

### 2.3. The Effect of Grinding Time on the Bioaccessibility of Antioxidant Properties and Phenolic Content

The bioaccessibility of phenolic compounds from carob products was recently studied [28,29,30], but the effect of grinding time has not yet been determined. Chait et al. [28] observed an increase in free phenolic compounds, but the degradation of bound and conjugated compounds was determined after complete in vitro digestion. Pure phenolic acids and flavonoids were degraded during the oral, gastric, and intestinal digestion process, but the authors found that bioaccessibility depended on the product type [29]. For example, TPC retention was similar for carob powder, syrup, fibre, and extract. However, higher retention of DPPH was observed for carob powder than for carob extract. The digestion liquid was used to examine the effect of various grinding times on the release of phenolic substances from carob powder after a three-stage in vitro digestion process in our study. We assume that phenolics in digestion fluid are readily absorbed into the small intestine. As can be seen in Table 3, luteolin and apigenin were the flavonoids most affected by the digestion process of carob powder samples in this study. Luteolin content decreased by 12.5–19.5 μg g^−1^, which is equivalent to 5–8% of its initial values. Apigenin content was reduced to 94–95%. Apigenin losses were similar to those observed in defatted lupin seeds during two-stage in vitro digestion [35]. Ferulic and cinnamic acid were the most stable during in vitro digestion showing 42–51% and 33–39% bioaccessibility. The grinding time strongly affected the content of vanillic acid (*p* < 0.001), cinnamic acid (*p* < 0.001), luteolin (*p* < 0.01), and apigenin (*p* < 0.001) after the three-stage digestion process, but no trend can be identified. Although the vanillic acid content was the highest in CP30, the maximum content values for cinnamic acid, luteolin, and apigenin were observed in different CP samples. Particle size did not influence the phenolic content after in vitro digestion. When the bioaccessibility of substances in carob powder samples is compared, we get a clearer overview of their fate during digestion. Bioaccessibility was remarkably different in the CP30 and CP60 samples. For example, 23% and 14% vanillic acid was observed after the digestion of CP30 and CP60 samples, respectively. A further increase in the grinding time resulted in retention similar to that of the CP60 sample. The same pattern was observed for ferulic acid (decrease from 51% to 40%) and naringenin (decrease from 29 to 12%). It should be noted that the level of bioaccessibility is dependent on the experimental procedure applied to determine the initial content. The in vitro digestion process takes place in an aqueous environment, so the extraction of phenolics into a water solution is a better option [36,37]. However, water/organic solution mixtures were also used for the determination of the initial content of phenolic compounds [38,39], which were then used for the bioaccessibility calculation.

The total phenolic (*p* < 0.01) and flavonoid (*p* < 0.05) content of carob powder after in vitro digestion was affected by the grinding time. Although CP60 and CP120 have the highest TPC values (5.51 ± 0.27 and 5.66 ± 0.33 mg GAE g^−1^, respectively), the highest TFC values were observed in the CP30 and CP60 samples. Catechin content was similar for all carob samples. Whereas bioaccessibility of phenolic individuals decreased after the digestion of carob powder ground for 30 s, meanwhile TPC, TFC, and CC increased to 109, 126, and 148%, respectively. This discrepancy can be explained by the subsequent release of other phenolic compounds after in vitro digestion process. An increase in total phenolic content and, at the same time, a decrease in some hydroxybenzoic acids after gastrointestinal digestion of soursop was reported [40]. Although a reduction in the content of vanillic acid, caffeic acid, and catechins in the *Matricaria recutita* flower was observed after duodenal digestion, the TPC value increased [41]. This was evidenced by increased rutin, quercitrin or quercetin content in their research. A further increase in grinding time resulted in a decrease in both TPC and TFC bioaccessibility, reaching their minimum level of 88% and 49% for the CP180 sample, respectively. The bioaccessibility of catechins also exhibited a gradual decrease with increased grinding time, but it was still improved in carob powder ground for the longest time. Antioxidant properties of digestive liquid ranged from 10.24 ± 0.47 to 12.17 ± 1.23 mg Trolox g^−1^ using a DPPH assay and from 13.97 ± 0.26 to 16.12 ± 1.43 mg Trolox g^−1^ using a FRAP assay. The bioaccessible phenolic, flavonoid, and catechin contents were not affected by the particle size of carob powder. It is likely that the bioaccessibility of phenolics can be influenced by smaller particles than those used in our study. Li et al. [42] observed that the bioaccessible phenolic content and antiradical activity of wheat bran increased in the powder fraction smaller than 19.16 μm. Our findings suggest that carob powder ground for 30 s represents a good source of bioactive substances.

## 3. Materials and Methods

### 3.1. Chemicals

All enzymes, bile extract, 1,1-diphenyl-2-picrylhydrazyl radical (DPPH), 2,4,6,-tris(2-pyridyl)-s-triazine (TPTZ), 6-hydroxy-2,5,7,8-tetramethylchroman-2-carboxylic acid (Trolox), ferric chloride, aluminium chloride, Folin-Ciocalteu reagent (2N), vanillin, gallic acid, quercetin, catechin, the analytical standards for UHPLC analysis (vanillic acid, ferulic acid, cinnamic acid, and naringenin, all with purity ≥ 97%), methanol Chromasolv^®^, and acetonitrile Chromasolv^®^ were purchased from Sigma-Aldrich (St. Louis, MO, USA). Luteolin and apigenin standards were obtained from Thermo Fischer Scientific (Heysham, Lancashire, UK). Other inorganic salts, hydrochloric acid, and sodium hydroxide were analytical grade (Lach-Ner, Neratovice, Czech Republic).

### 3.2. Preparation of Carob Powder Sample

The dried carob pods were purchased in a store (Naturway, Czech Republic). A portion (42.0–43.0 g) was pulverised in a vibratory grinder BVM-2 (Brio, Hranice, Czech Republic) for 30, 60, 90, 120, and 180 s. The temperature of the metal parts of the grinder was checked with an infrared thermometer (830-T1, Testo, Titisee-Neustadt, Germany) to ensure the same initial grinding temperature. The temperature was measured in five random locations. The powder was prepared in duplicate, followed by mixing both batches. The carob powder was collected into plastic tubes and stored at room temperature. A portion (3.0 g) of powder was mixed with 10.0 mL of 90% (*v*/*v*) methanol and 30.0 μL of formic acid [30]. Extraction was supported by 20 min of sonication (RK106 ultrasound bath, Bandelin Electronics, Berlin, Germany) followed by centrifugation at 2440× *g* for 20 min (ST4R-Plus MD, Sorvall, Thermo Scientific, Waltham, MA, USA). The aliquots were stored at −25 °C until analysis.

### 3.3. In Vitro Digestion of Carob Powder

The static in vitro digestion process was adopted from the study by Swieca et al. [43], which comprises oral, gastric, and intestinal digestion stages. The in vitro digestion process has begun with a mixture of carob powder (3.0 g) and 7.0 mL of saliva juice [0.8% NaCl, 0.24% Na_2_HPO_4_, 0.02% KH_2_PO_4_, 4.0% α-amylase (≥10 U mg^−1^), pH 6.75] under constant agitation (20/min) at 37 °C (BSK ET618, Lovibond, Amesbury, UK) for 10 min. The gastric phase was initiated by decreasing the pH to 1.2 (5.0 M HCl) and adding 5.0 mL of gastric juice [0.03 M HCl containing 0.012% pepsin (2500 U mg^−1^)]. After 120 min of agitation at 37 °C, the mixture was alkalised with 1.0 M NaOH to pH 7.0. The intestinal phase was started by adding 5.0 mL of intestinal digestion fluid [0.1 M NaHCO_3_ containing 0.85% bile extract, 0.14% pancreatin (4 × USP)] and incubated at 37 °C for 120 min. The supernatant was obtained by centrifugation (2440× *g*, 15 min) and stored at −25 °C for further investigation. The same procedure was carried out without the presence of carob powder. The digestion experiment was carried out in triplicate.

The effect of the in vitro digestion process on the antioxidant properties and the content of phenolic substances was expressed as a percentage change compared to the methanolic extract.

### 3.4. Particle Size, Colour, Water Activity, and Moisture Sorption Determination

The particle size distribution of carob powder (CP) was measured by Morphologi 4 (Malvern Panalytical, Malvern, UK) by dispersing dry powder (3.0 mm^3^) on a glass plate. Three magnifications (5×, 20×, 50×) were used for the measurement. The particle size was expressed as lower (D_10_) and upper (D_90_) decils and a median (D_50_) of cumulative weight. It means that, for example, 10% of particles are smaller than the value of D_10_ (software Morphologi, v. 10.10). A span was calculated according to the equation:

(1) $$\mathrm{Span}=\frac{D_{0.9}-D_{0.1}}{D_{0.5}}$$

The span reflects the uniformity of the particle size distribution (value approaches 0 for an ideally uniform particle size distribution. The measurements of particle size were done in duplicate.

The colour was determined using a d/8° geometry spectrophotometer (HunterLab, Reston, VA, USA) and a small area view reflectance port (9.5 mm). A white tile was used as a standard. The colour was expressed as CIEL*a*b* where L* represents parameters of darkness (0)–lightness (100), a* red (+)–green (−) parameter, and b* yellow (+)–blue (−) parameter [44]. Five repetitions for each sample were performed.

The water activity of the powder samples was measured using the AquaLab TDL instrument (Meter Group, Pullman, WA, USA) at 25 °C immediately after grinding.

Moisture adsorption of powder was determined using a dynamic vapour sorption system (DVS Intrinsic Plus, Surface Measurement Systems, Wembley, UK) at 25 °C. A small amount of powder (15.0 mg) was distributed on an aluminium weighing plate and hung on the wire connected to the ultrasensitive analytical balance (± 0.1 μg) in a closed chamber. Before analysis, the sample was exposed to a dry airflow (200 ccm, moisture < 0.1%) for 4 h to obtain dry mass. Adsorption was carried out in relative humidity (RH) from 0 to 80% in 5% increments. Changes in the mass of the sample were monitored at 30 s intervals until the equilibrium was reached (<0.002% dm/dt for 20 min) for each RH level. The results were expressed as the equilibrium moisture content (EMC) on a dry mass basis (mg g^−1^).

### 3.5. Antioxidant Properties of Carob Powder

Antioxidant activity was determined by absorbance changes in the methanolic solution of DPPH [45]. The DPPH assay was performed using 5.0 mL of DPPH methanolic solution (12.5 mg per 500 mL) and 300 μL of sample extract. After 10 min of incubation, a decrease in absorbance was observed at 517 nm (UV-2600, Shimadzu, Kyoto, Japan). The results were expressed as Trolox equivalent antioxidant capacity (TEAC) on a dry mass basis (mg g^−1^. Ferric reducing antioxidant power (FRAP) assay was performed after the reaction of the sample extract with TPTZ solution and FeCl_3_ in an acidic environment [46]. Briefly, the FRAP reagent mixture was prepared by mixing 20 mM FeCl_3_, 10 mM of TPTZ (in 40 mM HCl), and 0.3 M acetic buffer solution (pH 3.6) in a respective ratio of 1:1:10. The results were expressed as TEAC (mg g^−1^).

### 3.6. Phenolic Content in Carob Powder

#### 3.6.1. Spectrophotometric Assays

Total phenolic content (TPC) was estimated using a mixture of sample extract (1.0 mL), 5.0 mL of distilled water and 0.5 mL of Folin-Ciocalteu reagent. After 5 min, 1.0 mL of 5% Na_2_CO_3_ was added, followed by 60 min incubation in a dark place. The increase in absorbance was monitored at 765 nm, and the results were expressed as gallic acid equivalent (GAE) per gram on a dry mass basis (mg g^−1^) [6].

The determination of total flavonoid content (TFC) followed this procedure [47]: 2.0 mL of sample extract with 1.0 mL of 2.0% AlCl_3_, distilled water, 1.0 M HCl and 1.0 M CH_3_COONa. The absorbance (425 nm) was read after 10 min incubation at laboratory temperature in a dark place. Quercetin was used as the standard (QUE/(mg g^−1^)). For catechin content (CC), the reaction mixture consists of 1.0 mL of sample extract and 2.5 mL of vanillin (4%) and sulfuric acid (both 25% methanolic solutions). Absorbance was read at 500 nm after 15 min of incubation. The result was expressed as catechin equivalent on a dry mass basis (CAT/(mg g^−1^)) [48].

#### 3.6.2. HPLC Analysis of Phenolic Individuals

Analyses were performed using the Agilent 1290 Infinity liquid chromatography system (Agilent Technologies, Santa Clara, CA, USA) consisting of the degassing unit, high-pressure pump, autosampler, column thermostat, and diode array detector. Chromatographic column Kinetex XB-C18 100 Å (150 × 2.1 mm, particle size 1.7 μm) was chosen for separation. The conditions for the separation and quantification of target compounds were adopted from our previous study [30]. Briefly, the mobile phase was formed by water acidified with formic acid (pH ~ 3.1; solvent A) and acetonitrile (solvent B). Gradient elution (0 min—3% (B), 34 min—12% (B), 55 min—30% (B), 60–70 min—90% (B)) was applied. Conditions were set as follows; injection volume of 2 μL, mobile phase flow rate of 0.4 mL min^−1^, a temperature of 40 °C, and detection wavelengths of 270, 290, and 320 nm according to the absorption maxima of separated compounds. Calibration standard solutions of phenolic acids (vanillic, ferulic, cinnamic, protocatechuic, caffeic, chlorogenic, syringic, p-coumaric) and flavonoids (rutin, luteolin, apigenin, naringenin) were prepared by dissolving analytical standards in 90% (*v*/*v*) methanol including 0.3% (*v*/*v*) of formic acid. Before analysis, all samples were filtered through a 0.22 μm PTFE syringe filter.

### 3.7. Statistical Analysis

The results were expressed as mean with standard deviation. The grinding time represents a factor in the 1-way analysis of variance (ANOVA) to determine its effect on the variables. Duncan’s test was applied for multiple pairwise comparisons between means. The correlation coefficient (*r*) was calculated to find the association between the variables. All tests were performed at the probability level *p* = 0.05 using Statistica software (v. 12.0, StatSoft, Tulsa, OK, USA).

## 4. Conclusions

The carob powder prepared by vibratory grinding had different properties in relation to the grinding time. However, increasing the grinding time did not lead to a pronounced decrease in particle size. The carob powder contained small particles after 60 s of grinding; then, an increase was observed when ground for 180 s. It should be noted that large particles were also found in the carob powder ground for a longer time, probably because of their agglomeration. Moisture adsorption corresponded to the particle size. The colour changes of the carob powder were found to be important with respect to the grinding time, but small differences were observed. As expected, the extraction ability of phenolics to methanol/water solution has increased, particularly from carob powder ground for 180 s. In contrast, the bioaccessibility of the phenolic substances in that sample was low. This can be explained by the destruction of bioactive compounds in an alkaline environment during intestinal in vitro digestion. Based on the results, we have to admit that longer grinding time has a negative effect on the bioaccessibility of phenolics, particularly flavonoids. It is sufficient to grind the carob pod for 30 s, ensuring good availability of nutraceuticals and lower energy cost for grinding. Since the particle size did not significantly influence the bioaccessibility of the phenolics, the effect of temperature during grinding or the microstructure of the particles can be further studied.

## Figures and Tables

**Table 1:** Particle size (µm), water activity (a_w_), and colour (CIEL*a*b*) of carob powder (CP) after vibratory grinding.

	Carob Samples					
	CP30	CP60	CP90	CP120	CP180	
D_10_	17.8 ± 1.1 ^b^	15.2 ± 0.5 ^c^	16.3 ± 2.1 ^bc^	21.4 ± 0.8 ^a^	19.4 ± 0.3 ^a^	**
D_50_	141.3 ± 2.5 ^a^	87.9 ± 1.7 ^d^	110.1 ± 3.5 ^c^	129.7 ± 2.9 ^b^	135.1 ± 1.8 ^b^	***
D_90_	427.7 ± 12.5 ^d^	572.7 ± 8.1 ^b^	494.6 ± 7.9 ^c^	810.6 ± 21.3 ^a^	429.7 ± 5.6 ^d^	**
Span	2.9	6.3	4.3	6.1	3.0	
a_w_	0.380 ± 0.005 ^d^	0.374 ± 0.002 ^d^	0.403 ± 0.001 ^c^	0.417 ± 0.001 ^b^	0.433 ± 0.003 ^a^	***
L*	47.1 ± 0.5 ^d^	51.1 ± 0.4 ^a^	49.4 ± 0.2 ^b^	48.0 ± 0.3 ^c^	47.0 ± 0.3 ^d^	***
a*	6.3 ± 0.4 ^bc^	5.8 ± 0.6 ^c^	6.3 ± 0.3 ^bc^	6.8 ± 0.4 ^ab^	6.9 ± 0.2 ^a^	***
b*	19.0 ± 0.5 ^b^	17.9 ± 0.4 ^c^	18.7 ± 0.3 ^b^	20.1 ± 0.4 ^a^	19.8 ± 0.3 ^a^	***

Results are expressed as mean ± standard deviation (particle size N = 2; a_w_ N = 3; colour N = 5). Different letters in superscript indicate significant differences in a row according to Duncan’s multiple pairwise test (*p* < 0.05). The significant effect of grinding time using ANOVA is marked as ** = *p* < 0.01, *** = *p* < 0.001. D_10_, D_90_, and D_50_ = lower decil, upper decil, and a median of the cumulative weight of the particle size distribution. CP30, CP60…CP180 represent carob powder ground for 30, 60…180 s, respectively.

**Table 2:** Phenolic content and antioxidant properties of carob powder (CP) prepared by vibratory grinding at various processing times.

	Carob Samples					
	CP30	CP60	CP90	CP120	CP180	
*w/(*μg g^−1^)						
Vanillic acid	3.95 ± 0.40 ^ab^	4.17 ± 0.40 ^ab^	3.58 ± 0.43 ^b^	4.08 ± 0.26 ^ab^	4.41 ± 0.08 ^a^	n.s.
Ferulic acid	8.64 ± 0.04 ^d^	10.77 ± 0.22 ^b^	10.85 ± 0.43 ^b^	10.09 ± 0.51 ^c^	11.28 ± 0.09 ^ab^	***
Cinnamic acid	45.20 ± 1.20 ^c^	50.30 ± 1.13 ^b^	47.80 ± 1.64 ^bc^	50.00 ± 2.15 ^b^	54.28 ± 1.42 ^a^	***
Luteolin	13.62 ± 1.82 ^b^	16.11 ± 1.44 ^b^	15.17 ± 1.74 ^b^	16.97 ± 1.00 ^b^	20.52 ± 0.57 ^a^	**
Naringenin	2.76 ± 0.06 ^c^	6.77 ± 0.25 ^ab^	6.67 ± 0.44 ^b^	7.28 ± 0.30 ^a^	7.28 ± 0.13 ^a^	***
Apigenin	1.29 ± 0.19 ^d^	1.94 ± 0.05 ^c^	2.17 ± 0.06 ^ab^	1.97 ± 0.11 ^bc^	2.27 ± 0.04 ^a^	***
*w*/(mg g^−1^)						
TPC as GAE	4.73 ± 0.21 ^d^	5.84 ± 3.98 ^ab^	5.41 ± 2.19 ^c^	5.72 ± 0.20 ^b^	6.07 ± 0.15 ^a^	***
TFC as QUE	0.19 ± 0.15 ^b^	0.33 ± 0.13 ^a^	0.27 ± 0.14 ^a^	0.32 ± 0.07 ^a^	0.39 ± 0.05 ^a^	n.s.
CC as CAT	0.33 ± 0.13 ^a^	0.36 ± 0.03 ^a^	0.35 ± 0.12 ^a^	0.44 ± 0.06 ^a^	0.46 ± 0.19 ^a^	n.s.
DPPH as TEAC	9.29 ± 3.16 ^b^	11.91 ± 0.51 ^a^	9.80 ± 0.53 ^b^	9.59 ± 1.62 ^b^	11.93 ± 0.74 ^a^	*
FRAP as TEAC	13.78 ± 1.33 ^c^	16.11 ± 1.80 ^b^	13.12 ± 1.28 ^c^	16.95 ± 1.08 ^ab^	16.52 ± 0.65 ^a^	***

All results were on a dry mass basis and expressed as mean ± standard deviation (N = 3). Different superscript letters indicate significant differences in a row according to Duncan’s multiple pairwise test (*p* < 0.05). The significant effect of grinding time using ANOVA was marked as * = *p* < 0.05, ** = *p* < 0.01, *** = *p* < 0.001, n.s. = not significant, TPC = total phenolic content, GAE = gallic acid equivalent, TFC = total flavonoid content, QUE = quercetin equivalent, CC = catechin content, CAT = catechin equivalent, DPPH = 1,1-diphenyl-2-picrylhydrazyl radical assay, FRAP = ferric reducing antioxidant capacity, TEAC = Trolox equivalent antioxidant capacity, CP30, CP60…CP180 represent carob powder ground for 30, 60…180 s, respectively.

**Table 3:** Phenolic content and antioxidant properties of carob powder (CP) after in vitro digestion, and its bioaccessibility.

	Carob Samples					
	CP30	CP60	CP90	CP120	CP180	
*w/(*μg g^−1^)						
Vanillic acid	0.91 ± 0.01 ^a^ (23) ^†^	0.57 ± 0.03 ^b^ (14)	0.56 ± 0.00 ^b^ (15)	0.55 ± 0.01 ^b^ (13)	0.67 ± 0.23 ^b^ (12)	***
Ferulic acid	4.45 ± 2.12 ^ab^ (51)	4.28 ± 0.17 ^b^ (40)	4.68 ± 0.28 ^ab^ (43)	4.65 ± 0.24 ^ab^ (46)	4.71 ± 0.18 ^a^ (42)	n.s.
Cinnamic acid	17.69 ± 0.37 ^b^ (39)	18.34 ± 0.38 ^a^ (36)	17.35 ± 0.35 ^b^ (36)	16.61 ± 0.28 ^c^ (33)	18.02 ± 0.14 ^a^ (33)	***
Luteolin	1.15 ± 0.03 ^a^ (8)	1.16 ± 0.04 ^a^ (7)	1.16 ± 0.03 ^a^ (8)	1.05 ± 0.04 ^b^ (6)	1.05 ± 0.07 ^b^ (5)	**
Naringenin	0.80 ± 0.02 ^a^ (29)	0.81 ± 0.02 ^a^ (12)	0.83 ± 0.01 ^a^ (12)	0.83 ± 0.03 ^a^ (11)	0.80 ± 0.03 ^a^ (11)	n.s.
Apigenin	0.08 ± 0.00 ^c^ (6)	0.09 ± 0.01 ^c^ (5)	0.12 ± 0.00 ^a^ (6)	0.10 ± 0.01 ^b^ (5)	0.10 ± 0.01 ^b^ (5)	***
*w*/(mg g^−1^)						
TPC as GAE	5.10 ± 0.10 ^c^ (109)	5.51 ± 0.27 ^a^ (94)	5.09 ± 0.12 ^c^ (94)	5.66 ± 0.33 ^a^ (99)	5.32 ± 0.29 ^b^ (88)	**
TFC as QUE	0.24 ± 0.02 ^ab^ (126)	0.28 ± 0.05 ^a^ (85)	0.20 ± 0.01 ^b^ (74)	0.23 ± 0.03 ^b^ (72)	0.19 ± 0.02 ^b^ (49)	*
CC as CAT	0.59 ± 0.23 ^a^ (148)	0.52 ± 0.18 ^a^ (141)	0.50 ± 0.13 ^a^ (143)	0.57 ± 0.18 ^a^ (129)	0.53 ± 0.16 ^a^ (116)	n.s.
DPPH as TEAC	10.24 ± 0.47 ^b^ (110)	12.17 ± 1.23 ^a^ (102)	11.21 ± 0.64 ^ab^ (114)	11.12 ± 1.16 ^ab^ (116)	11.09 ± 1.26 ^ab^ (93)	*
FRAP as TEAC	13.97 ± 0.26 (101) ^c^	16.12 ± 1.43 ^a^ (100)	15.07 ± 0.47 ^b^ (115)	16.05 ± 0.69 ^a^ (95)	14.70 ± 0.31 ^bc^ (89)	***

^†^ Bioaccessibility in % of the initial content (in brackets); different superscript letters indicate significant differences in a row according to Duncan’s multiple pairwise test (*p* < 0.05); the significant effect of grinding time using ANOVA was marked as * = *p* < 0.05, ** = *p* < 0.01, *** = *p* < 0.001, n.s. = not significant. TPC = total phenolic content, GAE = gallic acid equivalent, TFC = total flavonoid content, QUE = quercetin equivalent, CC = catechin content, CAT = catechin equivalent, DPPH = 1,1-diphenyl-2-picrylhydrazyl radical assay, FRAP = ferric reducing antioxidant capacity, TEAC = Trolox equivalent antioxidant capacity, CP30, CP60…CP180 represent carob powder ground for 30, 60…180 s, respectively.

## Data Availability

All the relevant data are only provided in the present paper.

## Supplementary Materials

The following supporting information can be downloaded at: https://www.mdpi.com/article/10.3390/molecules27227689/s1, Figure S1: Chromatogram of vibratory ground carob powder (CP180) extract.
