# Supplementary material for: The Effect of Vibratory Grinding Time on Moisture Sorption, Particle Size Distribution, and Phenolic Bioaccessibility of Carob Powder
Source: Molecules. 2022 Nov 9;27(22):7689. doi: 10.3390/molecules27227689 (PMC9698127; doi:10.3390/molecules27227689)
Supplement: Supplementary file 1 [file molecules-27-07689-s001.zip › molecules-1995344-supplementary.pdf]

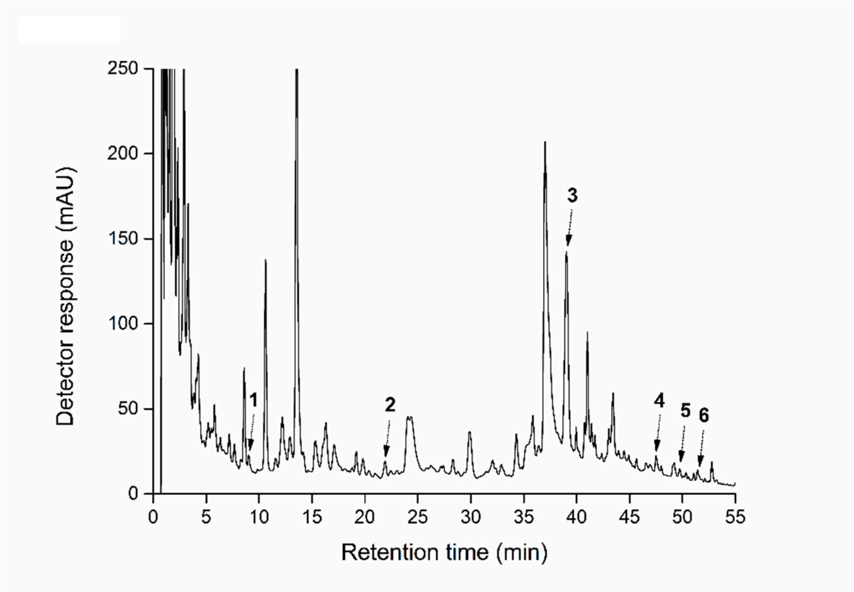

Figure S1. Chromatogram of vibratory ground carob powder (CP180). Kinetex XB-C18 100 Å (15 cm × 2.1 mm × 1.7 µm); mobile phase: (A) deionized water with formic acid addition (pH ~ 3.1) and (B) acetonitrile; gradient elution: 0 min – 3% (B), 34 min – 12% (B), 55 min – 30% (B), 60-70 min – 90% (B); injection volume of 2 µL; flow rate of 400 µL min^{-1}; temperature of 40 °C; detection at 270, 290 and 320 nm. Quantified compounds: vanillic acid (**1**), ferulic acid (**2**), cinnamic acid (**3**), luteolin (**4**), naringenin (**5**) and apigenin (**6**).
